# Distribution and Habitat Specificity of Potentially-Toxic *Microcystis* across Climate, Land, and Water Use Gradients

**DOI:** 10.3389/fmicb.2016.00271

**Published:** 2016-03-15

**Authors:** Sophi Marmen, Dikla Aharonovich, Michal Grossowicz, Lior Blank, Yosef Z. Yacobi, Daniel J. Sher

**Affiliations:** ^1^Department of Marine Biology, Charney School of Marine Sciences, University of HaifaHaifa, Israel; ^2^Department of Plant Pathology and Weed Research, ARO, Volcani CenterBet Dagan, Israel; ^3^Yigal Allon Kinneret Limnological Laboratory, Israel Oceanographic and Limnological ResearchMigdal, Israel

**Keywords:** cyanobacteria, distribution, *mcyD*, *mcyA*, microcystins, *Microcystis*

## Abstract

Toxic cyanobacterial blooms are a growing threat to freshwater bodies worldwide. In order for a toxic bloom to occur, a population of cells with the genetic capacity to produce toxins must be present together with the appropriate environmental conditions. In this study, we investigated the distribution patterns and phylogeny of potentially-toxic *Microcystis* (indicated by the presence and/or phylogeny of the *mcyD* and *mcyA* genes). Samples were collected from the water column of almost 60 water bodies across widely differing gradients of environmental conditions and land use in Israel. Potentially, toxic populations were common but not ubiquitous, detected in ~65% of the studied sites. Local environmental factors, including phosphorus and ammonia concentrations and pH, as well as regional conditions such as the distance from built areas and nature reserves, were correlated with the distribution of the *mcyD* gene. A specific phylogenetic clade of *Microcystis*, defined using the sequence of the *mcyA* gene, was preferentially associated with aquaculture facilities but not irrigation reservoirs. Our results reveal important environmental, geospatial, and land use parameters affecting the geographic distribution of toxinogenic *Microcystis*, suggesting non-random dispersal of these globally abundant toxic cyanobacteria.

## Introduction

Cyanobacteria, as part of a larger algal community, form the base of the food web in many aquatic environments (both marine and freshwater; Paerl and Paul, 2012). However, under appropriate conditions, many cyanobacterial species can grow at a rapid rate and form massive “blooms” which negatively impact water quality, especially when the blooming species produces toxins (Schinder and Valentyne, 2008; Paerl and Otten, 2013). Such blooms, termed “Harmful Cyanobacterial Blooms” (cyanoHABs), are one of the major threats to water quality worldwide, affecting many ecologically and economically important water bodies, and occasionally causing severe health problems and/or mortality in livestock and humans (Pouria et al., 1998; Carmichael, 2001; Brianda et al., 2003; Falconer and Humpage, 2005; Paerl and Huisman, 2009). In the United States alone, cyanoHABs have been estimated to inflict as much as $2 billion a year in losses from water made unfit for recreation, drinking or agriculture (Stone, 2011). Over the last several decades the frequency and severity of reported cyanoHABs has increased significantly, and toxic blooms have been observed in water bodies where they have not previously been recorded (Sukenik et al., 2012). To date, it is unknown whether these newly-observed blooms occur due to cyanobacteria that were always part of the microbial population (and potentially had bloomed but this was not reported) or whether these organisms have recently colonized the water body in question. If the latter is true, it is unclear whether the newly-introduced cyanobacteria migrated naturally (e.g., carried by the wind, animals or birds, Sukenik et al., 2012; van Leeuwen et al., 2012), or were introduced by man, for example through fish stocking or ballast water (Padilla and Williams, 2004).

The toxins that are most commonly found in freshwater environments worldwide, and potentially have the highest impact on water use, are microcystins (Carmichael, 2001; Valério, 2010). Microcystins are cyclic peptides (Valério, 2010), which are synthesized by a non-ribosomal peptide/polyketide synthase (NRPS/PKS) enzyme complex encoded in the highly conserved *mcy* gene cluster which comprises two operons (Dittmann and Börner, 2005; Vasconcelos et al., 2010). The large microcystin synthetase complex consists of peptide synthetases (*McyA–C*), a polyketide synthase (*McyD*) and hybrid enzymes (*McyE-G*) (Pearson et al., 2004; Campos and Vasconcelos, 2010). In mammals, the organ most affected by microcystins is the liver, and specifically the hepatocytes that contain the target protein phosphatases type 1 and 2 (Dittmann and Wiegand, 2006; Valério, 2010). High concentrations of microcystins in water have been directly linked to cases of intoxication and death in animals and humans, and they are also potential carcinogens (Carmichael et al., 2001; Hernández et al., 2009). Microcystins are most commonly produced by cyanobacteria of the genus *Microcystis*, although other genera (e.g., *Planktothrix, Oscillatoria, Anabaena, Anabaenopsis, Nostoc, Hapalosiphon, Snowella*, and *Woronichinia*) are also known to produce the toxin (Allender et al., 2009; Campos and Vasconcelos, 2010; Valério, 2010; Kurmayer et al., 2014).

The abundance and severity of cyanoHABs caused by *Microcystis* are rising worldwide, affecting millions of people (Carmichael, 2001; O'Neil et al., 2012). It is known that increases in nutrient load, temperature, salinity, and UV light may all contribute to the emergence of microcystin—producing cyanoHABs (Davis et al., 2009; Dziallas and Grossart, 2011; Paerl et al., 2011a; O'Neil et al., 2012). However, it is currently unclear whether all water sources contain toxic cyanobacteria, (Kurmayer et al., 2011; van Gremberghe et al., 2011), or whether the distribution is patchy, with some locations harboring toxinogenic populations and others not. It is also unclear whether the presence of such potentially-toxic populations is related to the conditions within the water body or the region surrounding it. Importantly, since cyanoHAB development requires the presence of cells capable of toxin biosynthesis either in the water body or in the sediment (Green et al., 2008; Tanabe et al., 2009), it is likely that the patterns of local distribution determine, at short time scales, where and when these blooms will occur.

To start answering these questions, we studied the distribution of *Microcystis* with the genetic capacity to produce microcystins in the water column of almost 60 different freshwater bodies across Israel. Despite its small geographic size, Israel is rich in different climatic and geographic regions: from Mediterranean climate (cool, wet winters and hot, dry summers) to desert (with an average annual precipitation of less than 25 mm), from highly urban to almost unsettled and from highly industrial to mainly agricultural or natural areas. Many small water sources, such as springs, irrigation reservoirs and aquaculture facilities are found within this tapestry of different local and regional environmental conditions (Supplementary Figure 1). Most of these water sources are relatively isolated, i.e., they are not directly connected to each other (e.g., through channels or streams). Similar conditions are found in many Mediterranean and semi-arid regions. The small size of the country and its conspicuous physical variation provide a unique natural laboratory for analyzing the effects of local and regional climate and land use on aquatic microbial communities. The goals of the study were: (1) to map the distribution of potentially-toxic *Microcystis* strains during the period of the year when blooms are most common (and thus cells most likely to be found in the water column), using a highly conserved fragment of the *mcyD* gene; (2) to characterize the environmental (local and regional) factors associated with the presence of toxin-producing strains in the water column, and, (3) to determine, using the phylogenetically-informative *mcyA* gene, whether toxinogenic strains in Israel belong to a single or multiple populations, each potentially associated with a specific aquatic niche.

## Materials and methods

### Collection of samples for molecular and meta-data analyses

**Figure 1:**
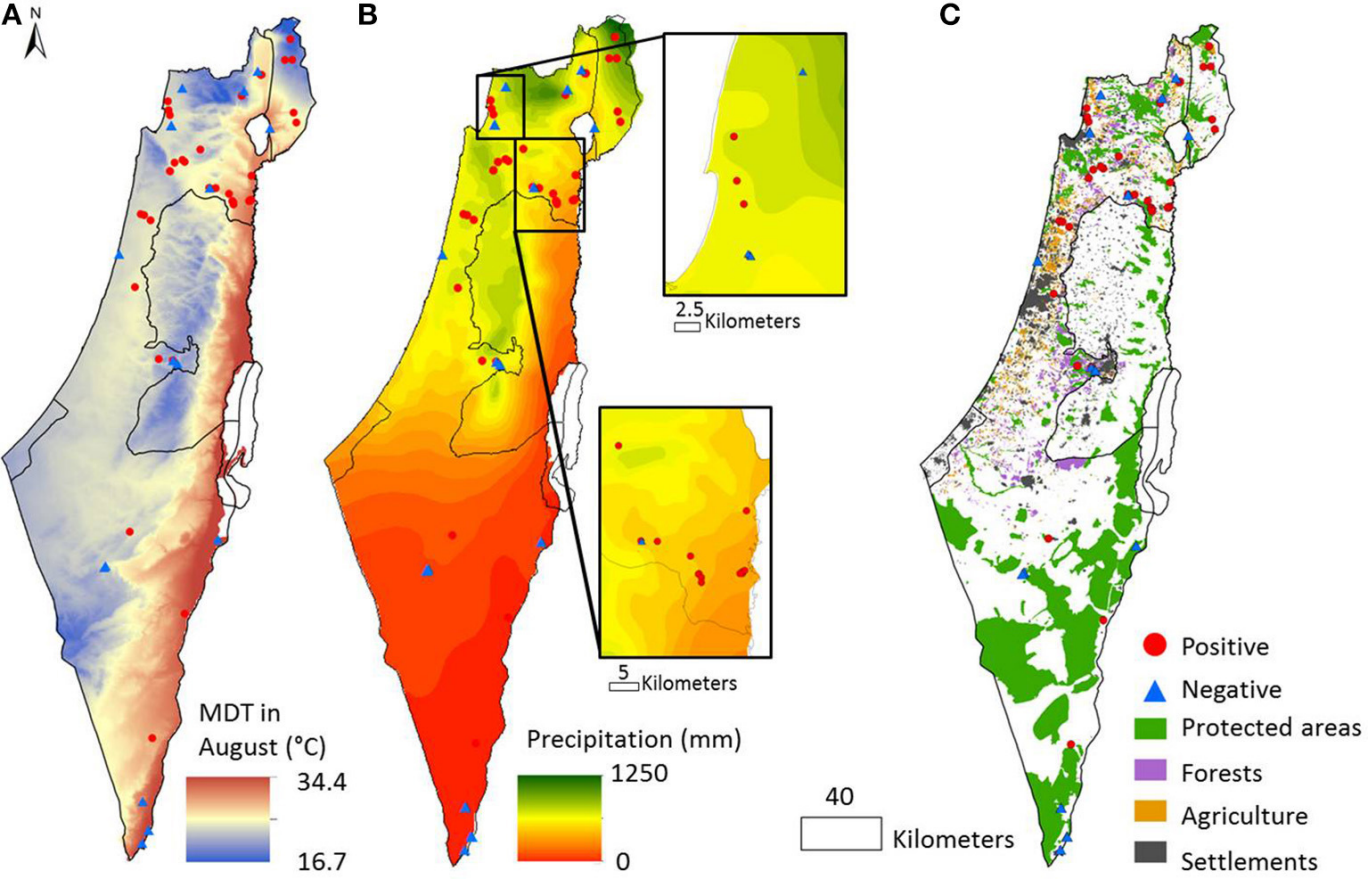
**Environmental gradients and the distribution of toxinogenic *Microcystis* across Israel**. Sampling locations where *mcyD* gene was detected are represented by a red circle, those where *mcyD* was not detected by a blue triangle. **(A)** Average day temperature range in August (mdt8); **(B)** Average annual precipitation; **(C)** Land use. Exact GPS coordinates of the sampling locations, as well as all of the parameters measured, are presented in Table 1.

A total of 58 water bodies were sampled across Israel (Figure 1, Table 1, Supplementary Figure 1). Most of the samples (51) were collected between July and the beginning of November of 2011, a period that was characterized by stable, hot and dry weather. During this period *Microcystis* blooms are often observed in small reservoirs around Israel, maximizing the possibility of detecting cells in the water column. Another, seven locations at the desert south of Israel were sampled during the following winter for technical reasons (rows 54–60 in Table 1, sampled during January and March, 2012). Each location was sampled once from the edge of the water body during the late morning to early afternoon. During sampling, dissolved oxygen, temperature and pH were measured using field probes (Eutech instruments, Singapore). At each sampling location, 5 l of surface water were collected. The collected water was filtered on GF/F filters (nominal pore size 0.7 μm, Whatman, UK) for DNA and particulate nutrients and on GF/C filters (1.2 μm) for chlorophyll extraction. DNA sample were overlaid by lysis buffer (50 mM Tris pH = 8.3, 0.75 M Sucrose, 40 mM EDTA). The filtrate from the GF/F filters was collected for dissolved nutrients analysis. Filtration was performed until the filters were blocked, and the volume of filtered water was recorded (Table 1). All filtration steps were performed within 1 h at the sampling site, using a hand-held vacuum pump (Mityvac, USA). All samples were placed in a cooler with dry ice, and transferred to a −80°C freezer (DNA and chlorophyll) and −20°C (dissolved nutrients) within 10 h. The filtration equipment was washed with ethyl alcohol (70%) and distilled water between sampling sites, to prevent any cross contamination.

**Table 1:** **Summarization of the sampled locations, ITM coordinates and all measured a-biotic parameters**.

**Name**	**Code**	**ITM coordinates**		**Elev (m)**	**mcyD detected? Yes = 1, No = 0**	**Water body type**	**Dissolved nutrients**					**A-biotic parameters**			**Vol**	**LOD (cells/mL)**	**Particulate nitrients**	
		**North**	**West**				**Phosphrus (μg P/L)**	**Ammonia (mg N/L)**	**Nitrite (mg N/L)**	**Nitrate (mg N/L)**	**N:P**	**PH**	**Temperature**	** Chl a (μg/ml)**			**mg N/L**	**mg C/L**
Birket Ram	BR	271526	793387	948	1	L	5.41	0.15	0.03	0.47	121.12	8.9	26.1	0.01	200	0.05	0.59	3.60
Poleg pool	BP	184916	684752	14	0	L	6.80	1.26	0.01	0.03	191.27	7.7	31.8	0.05	60	0.17	0.59	3.60
Gesher (North of Beit Shean)	Bsh-N	251953	724386	−224	1	Irr	N/A	N/A	N/A	N/A		7.7	30.1	0.00	150	0.07	N/A	N/A
Fish pond near Bitzat Zita	BZ	195838	704944	16	1	F.p	9.33	0.93	0.01	0.06	107.01	9.0	32	0.16	40	0.25	2.06	13.99
Shomrat reservoir	CS	209612	762054	15	1	Irr	20.13	0.03	0.00	0.01	1.93	8.8	18	0.42	15	0.67	5.76	33.30
Ga'aton Lake	DS	216781	768801	148	0	L	24.50	0.21	0.02	3.70	160.66	7.3	N/A	0.01	400	0.03	0.14	1.07
Ein Ovdat	EA	177303	526330	477	0	Spr	3.20	0.04	0.00	0.03	24.10	7.9	20.83	0.01	250	0.04	0.18	0.93
Ein Afek–Eshel pool	EA-Ash	211256	749824	6	0	S.sys	8.06	1.41	0.07	6.71	1016.29	7.4	28.5	0.00	100	0.10	N/A	N/A
Ein Afek–Big Lake	EA-Bl	211388	749712	7	0	S.sys	3.30	0.21	0.14	4.32	1417.91	7.8	29.6	0.01	100	0.10	N/A	N/A
Ein Afek–Metzuda pool	EA-MZ	211091	749905	6	0	S.sys	3.61	0.84	0.12	5.57	1810.60	7.8	31.9	0.01	50	0.20	N/A	N/A
Ein Afek - Tzaizer pool	EA-Zn	211404	749612	9	0	S.sys	3.43	0.87	0.06	6.44	2152.80	7.0	26.9	0.00	400	0.03	N/A	N/A
Ein Bihura	EBH	212167	631074	604	1	Spr	22.61	0.03	0.19	1.51	76.75	7.6	20	0.17	85	0.12	7.73	29.71
Ein Emi pool 1	EE-A	210693	726622	98	1	Spr	16.33	0.01	0.02	0.39	25.75	N/A	N/A	0.00	200	0.05	N/A	N/A
Ein Emi pool 2	EE-B	210685	726674	96	1	Spr	15.82	0.09	0.02	0.27	24.36	N/A	N/A	0.02	200	0.05	N/A	N/A
Ein Harod reservoir	EH	233828	718152	−20	1	Irr	86.92	0.08	0.01	0.32	4.72	N/A	N/A	0.02	100	0.10	N/A	N/A
Ein Hamifratz fish pond	EHM	209941	757464	0	1	F.p	402.55	0.07	0.17	0.07	0.78	8.8	16.8	0.22	50	0.20	3.32	14.49
Ein Israel reservoir	EI	230672	718212	96	0	Irr	11.85	0.01	0.04	6.68	568.26	7.0	22.4	0.01	200	0.05	N/A	N/A
Ein Israel fish pond	EIK	230471	718276	97	1	F.p	1124.05	0.44	1.16	4.60	5.52	8.5	29.1	0.18	80	0.13	N/A	N/A
Ein Kisalon	EK	205025	631636	545	1	Spr	195.54	4.19	0.06	0.20	22.80	7.4	23.6	0.00	500	0.02	0.11	0.77
Ein Lior	EL	246879	764711	759	1	Spr	10.67	0.04	0.06	7.45	706.33	7.6	N/A	0.00	500	0.02	N/A	N/A
Ein Muda (Park Hamayanot)	EM	242804	709830	−111	1	Spr	15.24	0.02	0.01	2.71	179.35	N/A	N/A	0.00	500	0.02	0.04	0.29
Ein Nevoria	EN	247951	767301	687	0	Spr	20.03	0.52	0.01	0.16	34.17	7.4	N/A	0.38	5	2.00	N/A	N/A
Yeruham reservoir	ER	190377	544294	452	1	L	49.79	0.63	0.23	1.60	49.44	7.5	20.90	0.00	250	0.04	0.09	0.63
Ein Sataf	ES	212183	631009	590	0	Spr	6.07	0.12	0.06	1.38	255.52	8.2	23.3	0.11	170	0.06	N/A	N/A
Ein Shokek (Park Hamayanot)	Esh	242182	711523	−95	1	Spr	8.98	0.19	0.01	3.01	356.56	6.9	N/A	0.00	400	0.03	0.04	0.25
Ein Sarig	ESR	214247	629442	733	0	Spr	448.68	8.33	0.86	0.74	22.13	8.1	23	0.19	100	0.10	2.16	11.59
Ein Tamar	ET	234718	540614	−365	0	Spr	8.08	0.03	0.01	0.04	9.21	7.0	26.90	0.00	700	0.01	N/A	N/A
Fish pond–Park Hamayanot	FP	242781	710810	−99	1	F.p	14.29	0.11	0.23	0.93	88.68	8.2	N/A	N/A	63	0.16	0.34	3.43
Hedera river 1	H1	197584	704406	20	1	Ri	178.51	0.35	0.05	0.08	2.68	9.2	N/A	0.02	125	0.08	N/A	N/A
Hedera river 2	H2	200825	701892	27	1	Ri	N/A	N/A	N/A	N/A		7.5	N/A	N/A	25	0.40	N/A	N/A
Kfar Masarik fish pond	KM	210642	755054	2	1	F.p	7.95	0.18	0.01	0.04	28.60	7.8	22.3	0.14	30	0.33	1.19	7.05
Kfar Yehushua fish pond	KY	212955	730952	28	1	F.p	812.48	0.04	0.00	0.02	0.07	8.1	32.6	0.04	75	0.13	N/A	N/A
Shaabania reservoir	MD	273138	756267	463	1	Irr	N/A	N/A	N/A	N/A		8.1	27.5	0.02	70	0.14	N/A	N/A
Reservoir flowing into Kinnert Lake	MDb	261222	748334	−198	0	Irr	N/A	N/A	N/A	N/A		8.2	30.7	0.01	100	0.10	0.24	1.46
Bab-El-Hawa Reservoir	MGr	272484	783069	950	1	Irr	322.02	0.19	0.02	0.13	1.06	8.2	26.3	0.05	25	0.40	0.61	5.53
Hispin reservoir	MH	274523	751262	423	1	Irr	6.65	0.09	0.00	0.00	14.41	8.4	28.7	0.02	70	0.14	N/A	N/A
Maoz Chaim fish pond 1	MH-EE	251116	711623	−231	1	F.p	163.90	0.15	0.28	1.17	9.76	N/A	N/A	0.08	60	0.17	N/A	N/A
Maoz Chaim fish pond 2	MH-EP	251078	711988	−236	1	F.p	8.08	1.58	0.18	4.71	799.86	7.3	29.1	0.05	100	0.10	N/A	N/A
Maoz Chaim fish pond 3	MH-LL	251594	712251	−238	1	F.p	31.58	0.01	0.17	0.53	22.48	8.2	30.2	0.13	35	0.29	N/A	N/A
Maoz Chaim fish pond 4	MH-N	250644	711630	−236	1	F.p	43.29	0.14	0.25	1.16	35.89	7.9	30.5	0.16	40	0.25	N/A	N/A
Orvim reservoir	MO	268820	782932	807	1	Irr	123.56	0.10	0.01	0.02	0.96	9.3	26.9	0.02	75	0.13	0.49	2.94
Ein Mor	MOR	177984	527124	394	0	Spr	7.75	0.08	0.01	0.04	15.80	7.5	21.00	0.00	250	0.04	0.06	0.53
Mekorot Hayarkon	MY	193030	668076	19	1	S.sys	6.88	0.44	0.01	0.44	128.93	7.2	24.5	0.00	200	0.05	N/A	N/A
Nahalal reservoer 1	NH1	218180	731394	59	1	Irr	398.15	0.02	0.01	0.01	0.10	7.9	31.2	0.02	100	0.10	N/A	N/A
Nahalal reservoer 2	NH2	216997	732408	59	1	Irr	288.51	0.24	1.64	9.58	39.72	8.0	29.6	0.08	50	0.20	N/A	N/A
Kibutzim River	NK	242263	711567	−97	0	Ri	8.73	0.28	0.01	2.84	357.85	7.1	N/A	0.00	150	0.07	0.07	1.15
Neot Smadar reservoir	NS	201804	440086	405	1	L	10.35	0.03	0.01	0.14	16.63	8.2	14.00	0.01	300	0.03	0.26	2.21
Sapir park	PS	218139	502958	−26	1	L	8.62	0.05	0.01	0.44	58.47	7.7	14.70	0.01	300	0.03	0.42	2.38
Sahne	SAH	240549	715175	−87	1	S.sys	8.66	0.06	0.06	3.67	437.58	7.6	29.9	0.00	200	0.05	N/A	N/A
Hula Nature Reserve–supply channel	SH1	256595	775338	64	1	Ri	275.63	0.01	0.01	1.30	4.81	N/A	N/A	0.06	45	0.22	0.69	5.73
Hula nature Reserve–lake	SH2	257020	775372	60	1	L	11.34	0.01	0.00	0.01	2.72	N/A	N/A	0.06	40	0.25	1.89	11.00
Einan river	SH3	254881	777254	70	0	Ri	24.12	0.03	0.01	1.86	78.87	N/A	N/A	0.00	280	0.04	0.14	1.04
Eilat saltern 1	SP1	199673	393452	26	0	Salt	N/A	N/A	N/A	N/A		8.0	19.40	0.00	300	0.03	0.33	2.30
Eilat saltern 2	SP2	199831	393393	17	0	Salt	N/A	N/A	N/A	N/A		8.2	17.40	0.00	120	0.08	0.58	2.38
Eilat saltern 3	SP3	196501	386852	8	0	Salt	N/A	N/A	N/A	N/A		8.2	17.70	0.00	70	0.14	0.77	4.52
Timna pool 1	Ti-1	196760	407995	191	0	L	12.64	0.17	0.01	0.31	39.25	8.0	11.00	0.00	600	0.02	0.07	0.63
Timna pool 2	Ti-2	196743	408020	191	0	L	N/A	N/A	0.02	0.07		8.3	13.60	0.01	100	0.10	0.45	3.75
Tzipori	Zn	225952	737608	222	1	Ri	9.39	0.02	0.02	8.60	920.29	7.1	21.8	0.00	100	0.10	N/A	N/A

*Elev, elevation (in meters); LOD, limit of detection; Vol, volume filtered onto GF/F filters; Chl a, chlorophyll a concentration; F.p, fish pond; Irr, irrigation; L, lake; Spr, spring; S.sys, spring system; Ri, river; Salt, salterns*.

### Nutrients and chlorophyll analyses

The concentration of dissolved nutrients was determined using a colorimetric standard method (APHA, 2005) in a flow injection automated ion analyzer (Quikchem, 8000 LACHAT instruments). The concentration of phosphorus was determined by the MAGIC method (Karl and Tien, 1992). Filters for particulate nutrient analysis were dried overnight at 60°C, weighed and analyzed using a C/H/N analyzer (Perkin Elmer). All of the nutrient measurements were performed at the Kinneret Limnological Laboratory, Israel.

Extraction of Chlorophyll was performed in absolute methanol for 12 h at room temperature in the dark and the extract filtered through a 0.2 μm filter. Chlorophyll *a* (Chl *a*) concentration was determined spectrophotometrically (Ritchie, 2008).

### Environmental DNA extraction, PCR assays, and cloning procedure

Genomic DNA extraction was performed using a previously published protocol (Massana et al., 1997) with several modifications (Tzahor et al., 2009).

To determine whether there are toxic *Microcystis* species in the sampled waters, a sensitive end-point Polymerase Chain Reaction (PCR) was performed using MSF/R (Tillett et al., 2001) and mcyDF2/R2 (Kaebernick et al., 2000) primers sets for *mcyA* and *mcyD* genes respectively (Supplementary Table 1). The *mcyD* primers amplify the relevant genes from *Microcystis* but not from other cyanobacterial genera, as determined by two approaches: (1) BLAST against cyanobacterial genomes in the Integrated Microbial Genomes (IMG) server (Markowitz et al., 2014); (2) cloning and sequencing eight PCR fragments from four locations, all of which produced sequences which clustered together with *mcyD* from *Microcystis aureginosa* in a phylogenetic tree (Supplementary Figure 2). The *mcyD* primers were more sensitive than the *mcyA* primers, with a limit of detection of 10 toxinogenic cells/filter for *mcyD* and 100 toxinogenic cells/filter for *mcyA*, (Supplementary Figure 3), but were not phylogenetically informative (compare Supplementary Figures 2, 5). The *mcyA* primers were also *Microcystis*-specific, as shown in **Figure 5** (see also Tillett et al., 2001). The end-point PCR for *mcyD* was at least as sensitive as quantitative PCR, with an average limit of detection of ~160 cells/L compared to ~260–400 cells/L in other studies (Rinta-Kanto et al., 2005; Baxa et al., 2010). For these reasons, and because our study focuses on identifying where and when potentially toxic cells are found in the water body rather than on the relationship between the quantity of toxinogenic strains and environmental conditions, we used end-point PCR with the *mcyD* primers for the identification of toxinogenic populations, and the *mcyA* gene for phylogenetic analyses. In all locations from which *mcyA* was amplified, *mcyD* amplification was also observed. As a positive control (e.g., to rule out PCR inhibitors), PCR was performed also using primers for general *16S* rRNA (Frank et al., 2008) and cyanobacterial *16S* rRNA genes (Nübel et al., 1997). Full details of the PCR program, primer sequences and enzymes used are detailed in the Supplementary Experimental Procedures. The *mcyA* and *mcyD* sequences were deposited in GenBank under accession numbers KU867658 - KU867777.

### Phylogenetic analysis

One hundred and twenty *mcyA* sequences, obtained from 17 different water reservoirs, were aligned using ClustalW in MEGA5 (Tamura et al., 2011) and a maximum likelihood tree with 1000 bootstraps was constructed. The tree was visualized with ITOL (http://itol.embl.de/; Letunic and Bork, 2011), and two datasets of explanatory variables were added: the location of the sample and the water body type.

### Geographic analyses

To determine whether there are regional environmental factors that may be used as predictive variables for the presence of toxinogenic *Microcystis* population, we analyzed seven environmental parameters: elevation, mean daily temperature of the hottest month (August—mdt8), mean annual rainfall, distance from built areas (including cities, villages, army bases, and industrial zones), distance from forests, distance from natural protected areas and distance from agricultural fields. Elevation was derived from the digital elevation model (DEM) at 33-m resolution using ArcGIS (ESRI, Redlands, CA). To test whether the sampled locations that were found positive to the presence of *mcyD* gene differed from the negative locations in these parameters, we used Mann–Whitney *U*-test with Bonferroni correction. SPSS was used for all statistical analysis (version 21.0).

### Statistical analyses

All multivariate analyses were performed with R i386 2.15. The abiotic characterization of 35 sampling locations, for which the full suite of metadata was available (Table 1), was analyzed here with 2D non-metric multidimensional scaling (nMDS) ordination using measurements of phosphorus, ammonia, nitrite, nitrate, pH, temperature, and chlorophyll-*A* concentrations. The data were first standardized, and then log10-transformed prior to nMDS analysis. We used Bray-Curtis for the dissimilarity distances matrix. The nMDS has a stress value which represents the divergence of the real value from the ordination output (Warwick and Clarke, 1993). Stress values lower than 0.2 mean that the ordination is useful, but higher values (>0.1) should be analyzed with caution. With this analysis, grouping was performed according to the geography, water-body type (category), and presence or absence of the *mcyD* gene. One-way ANOSIM (Analysis of Similarities) was performed to statistically differentiate the abiotic characterization of each grouping. This test is a-parametric and does not assume normality of the data. The calculated test statistic R has a value between −1 and 1 and practically rarely goes below 0 (Warwick and Clarke, 1993). *R* = 1 means that all the repeats within a group are similar to each other rather than to repeats in other groups. When *R* = 0, the similarity within and among all groups is averagely the same. SIMPER (Similarity Percentages) identify the “important” component from all the abiotic factors; i.e., what is the relative contribution of each abiotic factor to the dissimilarity between all inter-group pairs of samples.

To find whether there are associations between different nutrient concentrations or abiotic conditions and the presence of *mcyD* gene, we used chi square (χ^2^) test (α < 0.05). In order to determine whether specific water body types are over- or under-represented in different clades of the phylogenetic tree, we performed a two-sided Fisher's Exact Test with Bonferroni correction using the free online GraphPad software (http://graphpad.com/quickcalcs/contingency1.cfm). To determine whether clades were associated with differences in the concentration of nutrients or chlorophyll, a Kruskall-Wallis test was performed in SPSS.

To relate the environmental variables to the binary dependent variable (presence/absence of the *mcyD* genes), we used logistic regression in the framework of Generalized Linear Models (GLMs). Full details of the GLM procedure are found in the Supplementary Methods.

## Results

### Characterization of sampling sites

**Figure 2:**
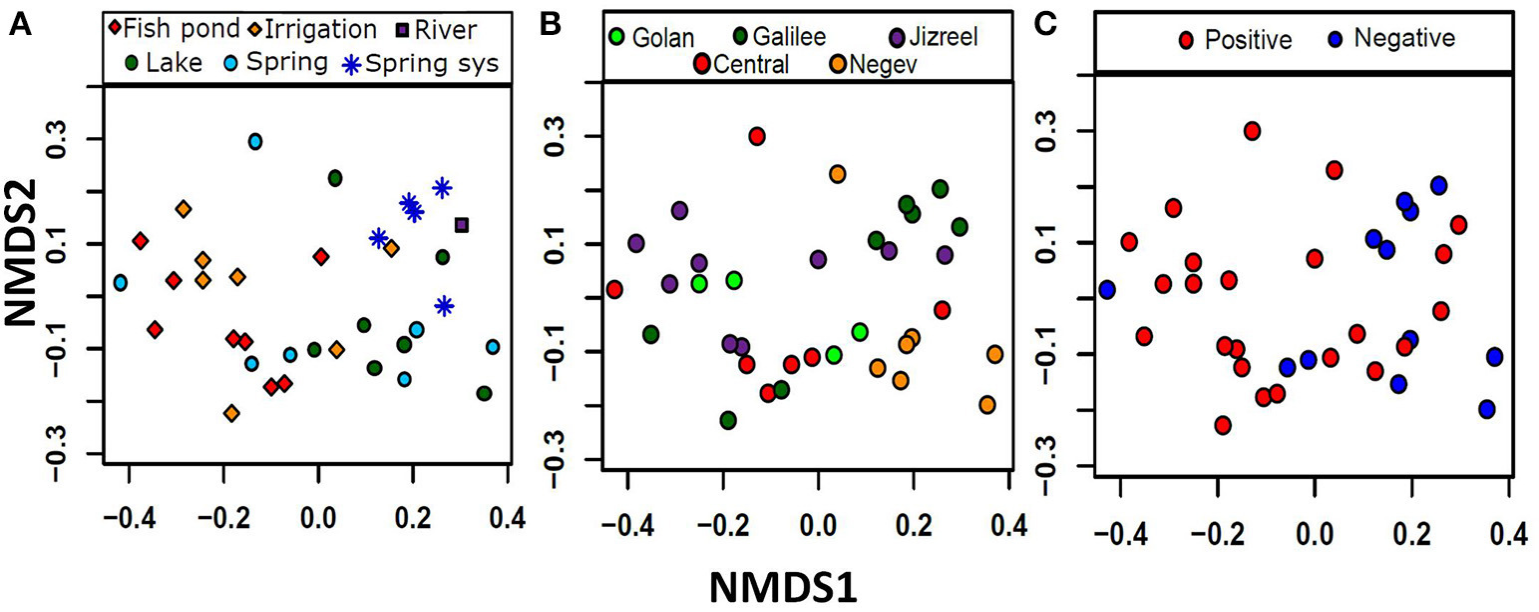
**nMDS multivariate analysis indicates grouping of water bodies by geography, water use and *mcyD* gene presence**. The samples ordinate to some extent by both the category or intended use of the water body **(A)** and the geographic location **(B)**. A statisticaly significante (*P* = 0.011) separation could also be observed between samples in which *mcyD* gene presence was observed and those in which is the gene was not detected **(C)**. The nMDS had a stress value of 0.149.

To map the distribution of potentially toxic *Microcystis* across different environmental conditions in Israel, we sampled 58 different water bodies belonging to seven functional categories: fish ponds, irrigation reservoirs, lakes, natural springs, spring systems (e.g., systems of several natural springs which flow from one into another), rivers and salterns (e.g., ponds for salt production). The sampling locations were selected in order to represent a wide diversity of environmental conditions (some of which are shown in Figure 1), and, for most locations, without prior knowledge of past cyanoHABs. The abiotic factors (e.g., pH values, phosphorus, ammonia, nitrate, and nitrite concentrations), which are expected to have a direct effect on the microbial community, differed widely between the sampled water bodies: pH values ranged from 6.9 to 9.3, phosphorus concentrations ranged from 3 to 1124 μg L^−1^, and total nitrogen concentration spanned from 0.03 to 11.5 mg L^−1^ (Table 1). Non-metric multidimensional scaling (nMDS) analysis of 35 locations for which a full suite of data were available (Table 1) revealed some grouping of the water bodies by both category (i.e., aquaculture, irrigation reservoirs or nature conservation), and geography (Figures 2A,B) with stress value of 0.149. Two of the categories of natural waters, namely spring systems and lakes, were each different from the agricultural waters (irrigation and fish-ponds, ANOSIM, Global *R* = 0.32, *p* < 0.001, pair-wise ANOSIM, *R* = 0.39–0.56, *p* < 0.01) mainly by the criteria of Chl *a*, phosphorus and nitrate values (Simper analysis). In terms of geography, the samples from the Negev Desert were different from the Jizreel Valley (Global *R* = 0.21, *p* = 0.003, pair-wise ANOSIM, *R* = 0.56, *p* < 0.01) and, with lower statistical support (*R* = 0.29–0.46, *p* < 0.05), from the other regions as well (Golan heights, Galilee, and Central region, Figure 2B). The main parameters for these variances were Chl *a*, phosphorus, nitrate and temperature values, as determined by Simper analysis. This may be due to the different sampling season (winter in the desert, summer in all other locations) or to inherent differences between the water bodies in the two climatically-different regions.

### Presence of potentially-toxic *Microcystis* cells in the water column of most sampled water bodies

We next determined whether potentially-toxic *Microcystis* were present in the water column of the locations described above. Using the *Microcystis*-specific PCR assay we found that th*e mcyD* gene (amplified using *Microcystis*-specific *mcyD* primers, see Materials and Methods) was detected in 64% (37/58) of the sampling locations (Figure 1, Table 1). Consistent amplification of *mcyD* genes was obtained from 10 cells/filter or more, representing an approximate limit of detection of one toxinogenic cell ml^−1^ depending upon the volume filtered at each sampling location (Table 1, Supplementary Figure 3A). Importantly, we re-visited nine sampling locations from which *mcyD* genes could not be amplified 4 years after the initial samples were taken (June 2015), collecting samples from the water column as well as the sediment. In six of these nine locations we still could not amplify the *mcyD* gene in the water column, with the other three locations all being part of an interconnected set of springs (Ein Afek, Table 1). Nevertheless, the *mcyD* gene could be amplified from all sediment samples. This suggests that, despite the presence of potentially toxic cells in the sediment, in most of these locations low densities of potentially toxinogenic strains in the water column are the norm during summer. It is tempting to speculate that these locations are inherently less hospitable to the potentially-toxic *Microcystis* strains.

### Presence of *mcyD* genes and the relationships with environmental factors

**Figure 3:**
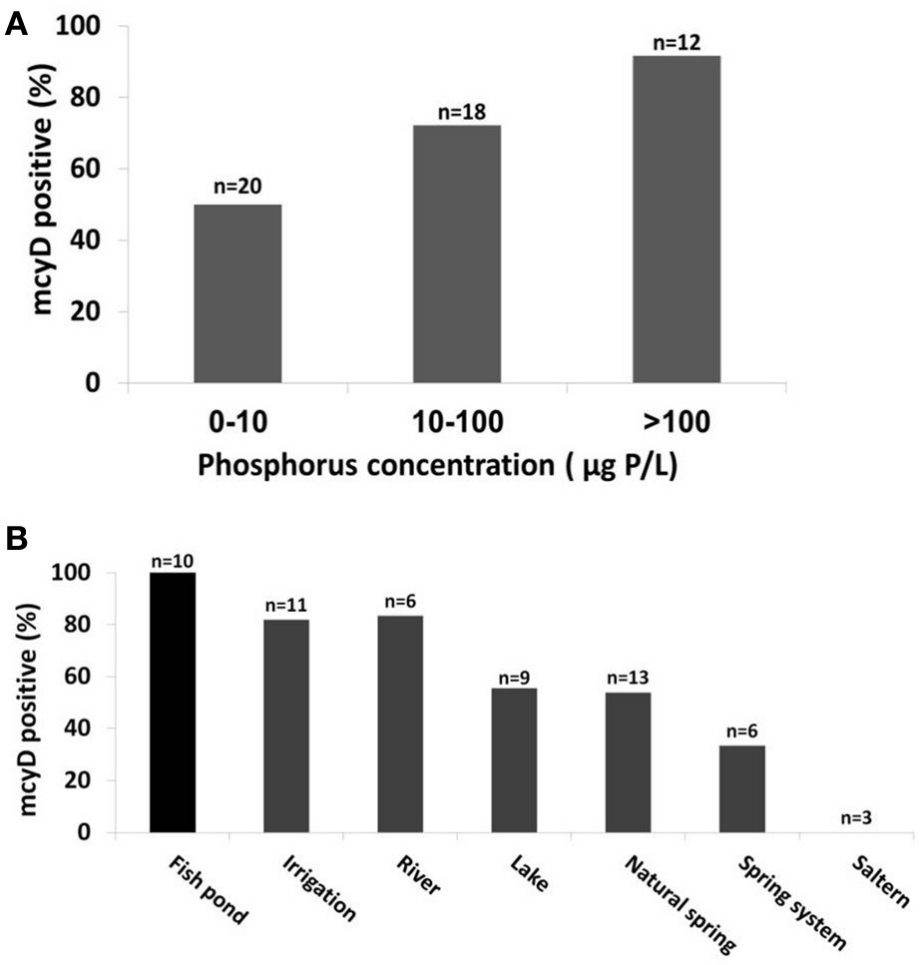
**The probability of *mcyD* being detected in the water bodies increases with phosphorus concentrations and in aquaculture facilities**. **(A)** A higher percent of *mcyD* gene presence was found in water with high P concentrations (a, *p* < 0.05). **(B)** Significant differences in the distribution of *mcyD* positive locations was observed among the different water body types (Kruskal-Wallis ANOVA analysis with *p* < 0.05). Note that all fish ponds contain toxinogenic *Microcystis* populations.

Many environmental factors are known to be associated with the presence or toxicity of *Microcystis* blooms, chief among them the concentrations of dissolved phosphorus and inorganic nitrogen (Vézie et al., 2002; Davis et al., 2009; Xu et al., 2010; Paerl et al., 2011b; Paerl and Paul, 2012). We therefore asked whether these factors are associated not only with the blooms or toxicity but also with the presence of potentially-toxic cells in general. As shown in Figure 3A, the probability of detecting the *mcyD* gene was significantly higher as the concentration of dissolved phosphorous increases. Furthermore, a negative trend was observed between the probability of *mcyD* detection and the N:P ratio, suggesting that the availability of phosphorus, rather than nitrogen, is correlated with, and potentially drives, the distribution of the *mcyD* containing strains (Supplementary Figure 4A). Moreover, when the presence/absence patterns of the *mcyD* genes were plotted on the nMDS analysis described above (Figure 2C), the *mcyD*-positive water bodies tended to cluster in the same area on the nMDS plot as the agricultural waters. All of the aquaculture water and most of the irrigation reservoirs contained potentially-toxic *Microcystis* strains, whereas only 50–60% of the natural water bodies (e.g., lakes and springs, the latter often collected into man-made pools, Supplementary Figure 1) contained potentially toxinogenic cells in sufficient concentrations for detection by the PCR assay (Figure 3B). In order to determine whether the correlation with high phosphate concentrations is in fact due to a cross-correlation between phosphate and aquaculture (implying that something else in the aquaculture-related water is in fact responsible for the presence of potentially-toxic populations), we performed the same statistical analysis without including values from fish ponds. The results exhibited a similar trend, with *mcyD* still associated with high phosphorus levels (Supplementary Figure 4C).

### The effect of local and regional conditions on the probability of *mcyD* presence

**Figure 4:**
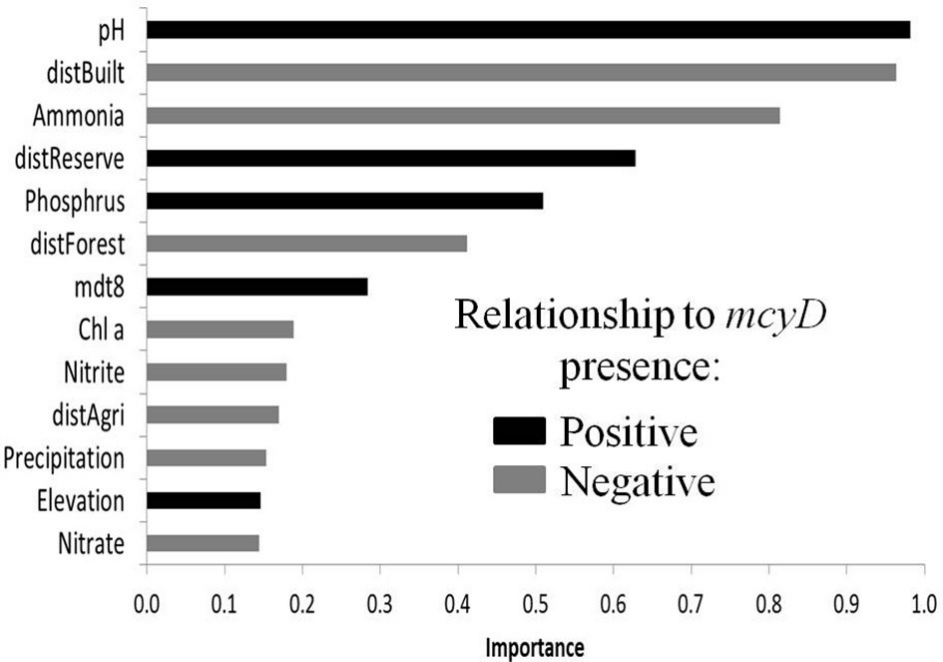
**The relative importance of different local and regional parameters using GLM**. The results of the same analysis performed with cross correlated in a distribution model of *mcyD* genes produced parameters removed and for locations found in the north of Israel are shown in Supplementary Table 3, and the coefficients relating each parameter to the presence/absence of *mcyD* are shown in Supplementary Table 4.

We next asked, are there additional factors besides nutrient concentrations that are correlated with a high probability of *mcyD* detection? To answer this question, taking into account not only the conditions within the water body but also those prevalent in the region surrounding it (up to several tens of kilometers), we superimposed our data onto geographically resolved maps of the mean summer day temperature during August (mdt8, Figure 1A), precipitation (Figure 1B) and elevation (Supplementary Figure 1). In addition, we used a composite map describing the boundaries of four categories of land use: forests, agricultural land, built areas and nature reserves (Figure 1C, Materials and Methods). Due to the limitations of the publically-available data, the latter map contains data for only ~37% of the total analyzed areas, and we therefore used the distance from the closest representative of each of these specific land-use categories for subsequent analyses. We used logistic regression in the framework of Generalized Linear Models (GLMs) to relate both local (e.g., abiotic parameters of the water body) and regional environmental variables to the presence of *mcyD* genes and determine the relative importance of each variable in the final distribution model (Supplementary Tables 2–4). Despite challenges associated with relationships between the explanatory variables and the differences between the desert south of Israel and the rest of the locations (see Supplemental Methods, Supplementary Figure 5), robust inferences can be drawn from this analysis: first, three local parameters, namely pH and the concentrations of ammonia and phosphorus, were designated as important model terms (Figure 4). The pH of the water bodies and phosphorus were positively related with *mcyD* presence (as shown by the coefficient of these parameters in the models, Supplementary Table 3) whereas ammonia revealed a negative relationship. Second, several regional parameters were also important in these models, specifically, the distance from built areas which was always inversely related to *mcyD* presence, suggesting that urban runoff may be a factor contributing to the presence of potentially-toxic cells in the water (Figure 4, Supplementary Table 3). Conversely, the distance from nature reserves was positively related to *mcyD* presence, suggesting that water bodies found in or around nature reserves have less of a chance to contain potentially-toxic populations. The regional inferences were weaker when the samples from the desert south of Israel were not included in the analysis, potentially due to the low density of built areas and forests in this region (Supplementary Figure 5).

### Phylogenetic distribution of potentially-toxic *Microcystis* populations among the sampling locations

**Figure 5:**
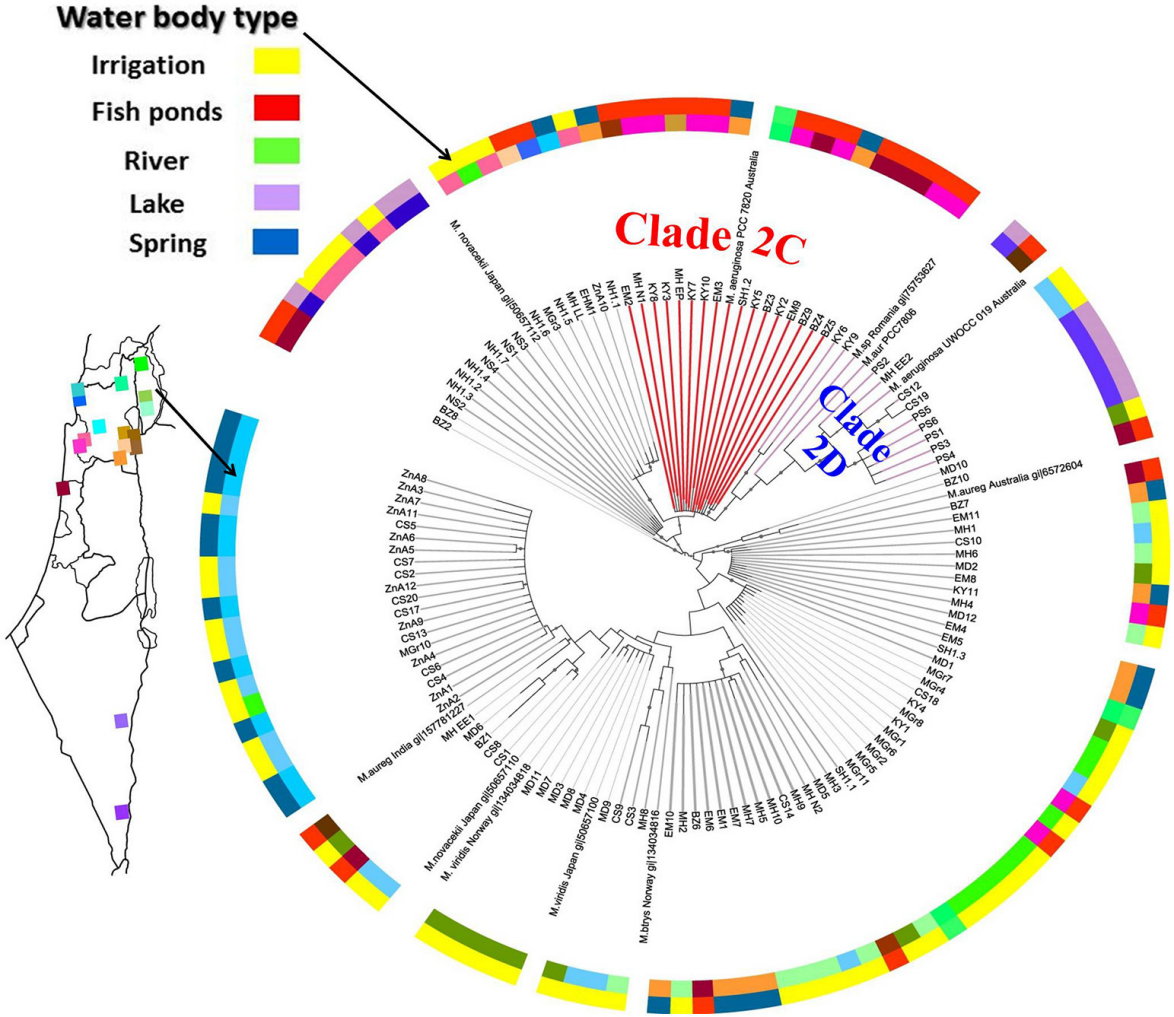
**A specific *mcyA* gene lineage is enriched in fish ponds and under-represented in irrigation reservoirs**. A maximum likelihood phylogenetic tree of the cloned *mcyA* genes, including also sequences from *Microcystis* isolates, is shown. Circles indicate branches with >50% bootstrap support. The tree is rooted at mid-point, sequences from other cyanobacteria (*Anabaena, Nostoc*, and *Planktothrix*) all clustered together as an outgroup (not shown). Color datasets on the rings surrounding the tree are the specific location of the sample (inner ring, with similar shades indicating geogrphically close locations) and the water body type (outer ring). Fish ponds are over-represented and irrigation reservoirs under-represented in the clade 2C (red, *p* < 0.01, fisher's exact test with Bonferroni correction). Lakes are over-represented in clade 2D (*p* < 0.05), however, most of the sequences from clade 2D came from the same lake and therefore this association requires further validation.

Given the significant differences in environmental conditions between the sampling locations, we next asked whether any genetic differences could be identified between the potentially-toxic populations inhabiting each water body, using the *mcyA* gene as a phylogenetic marker. No clear distance-decay pattern could be observed in the similarity of the *mcyA* gene assemblages (e.g., as shown using automatic ribosomal intergenic spacer analysis for stream bacteria, Lear et al., 2013), suggesting that the geographic distances between the sampled sites did not strongly affect the population structure of potentially-toxic *Microcystis*. This could also be due to the relatively small number of sampling locations and *mcyA* sequences (120 sequences from 17 locations). Nevertheless, as shown in Figure 5, some *mcyA* clades were preferentially associated with specific water body types. Specifically, one clade of *mcyA* was significantly associated with aquaculture facilities and negatively related to irrigation reservoirs (Figure 5, clade 2C, *p* < 0.01, Fisher's exact test with Bonferroni correction). No association was observed between specific clades and the concentrations of inorganic nitrogen phosphorus or Chl *a* (Krusal-Wallis test, *p*>0.05). This suggests that the potentially-toxic strains are not randomly distributed, and that the type of the water body or its intended use, rather than trophic state or the concentrations of specific nutrients, affect this distribution.

## Discussion

### What may influence the distribution of potentially toxinogenic *Microcystis*?

Harmful algal blooms in general, and those produced by *Microcystis* in particular, are on the increase. Elevated water temperature, increased salinity, stronger light intensity (especially UV), reduced mixing and higher nutrient loads may all provide a competitive advantage to *Microcystis* over other phytoplankton (Paerl and Huisman, 2008; Davis et al., 2009; Dziallas and Grossart, 2011; Van de Waal et al., 2011; Paerl and Paul, 2012). Many of the same environmental changes may also provide an advantage to toxic *Microcystis* strains (or other microcystin-producing bacteria) over non-toxic ones, although the mechanism (or the biological role of microcystins) is yet unclear (Davis et al., 2009; Dziallas and Grossart, 2011; Van de Waal et al., 2011; Kurmayer et al., 2014; Meissner et al., 2014). However, in order for a bloom to occur the water body in question must contain a viable “seed population” of toxinogenic strains, either in the water column or as resting stages in the sediment, that can respond to the favorable growth conditions. *Microcystis*, as a genus, has a global distribution, with strains isolated from all continents except Antarctica (van Gremberghe et al., 2011). Additionally, no clear phylogeographic patterns have been observed, suggesting few barriers to global dispersal (van Gremberghe et al., 2011; Moreira et al., 2013). Our results suggest that, at local to regional scales, potentially-toxic strains, identified through PCR of the *mcyD* gene, are common in the water column but not ubiquitous. Typically, planktonic *Microcystis* blooms are defined as >7 × 10^4^ cells L^−1^ (Baxa et al., 2010), and develop over a period of several weeks, from a starting planktonic population of at least >1000 cells L^−1^ (Davis et al., 2009). Given the sensitivity of the end-point PCR method we used (Supplementary Figure 3) and the typical rates of bloom formation and decline in nature (e.g., Davis et al., 2009; Baxa et al., 2010), it is likely that location in which *mcyD* genes were not detected by the PCR method, did not experience high *Microcystis* densities over the period of 2–3 months prior to our sampling, nor did such blooms occur over the following 2–3 months. However, we caution that such extrapolations are fraught with uncertainty, and cases have been recorded where blooms emerged quite rapidly (e.g., at the Antioch sampling site of the San Franscisco Estuary, toxic cell equivalents increased from just below our detection limit to ~2 × 10^7^ cells/L within less than a month and a half, Baxa et al., 2010).

The reasons for the observed “patchiness” in potentially-toxic *Microcystis* seems to be a complex interaction between many factors, including the intended use of the water (e.g., for aquaculture, irrigation etc.) and the location of the water body relative to urban centers and nature reserves. We suggest that these land- and water use parameters determine the concentration of inorganic nutrients and the pH of the water, which are the “proximal” drivers of potentially-toxic *Microcystis* distributions (Mattikalli and Richards, 1996; Crosbie and Chow-Fraser, 1999; Caccia and Boyer, 2005). As shown in Figure 4, Supplementary Figure 4B, and Supplementary Table 2, pH values and phosphorus concentrations were positively related to *mcyD* presence, whereas ammonia concentrations revealed a negative relationship. Inorganic phosphorus has previously been shown to be an important limiting factor for *Microcystis* growth (Nalewajko and Murphy, 2001; Xie et al., 2003; Paerl and Otten, 2013), and reduction of inorganic phosphorus in freshwaters has been suggested as a strategy to mitigate toxic blooms (Vézie et al., 2002). Our results extend this observation, suggesting that inorganic phosphorus also determines, to some extent, whether potentially-toxic populations inhabit a water body irrespective of whether or not they bloom. Notably, urban runoff often has very high inorganic phosphorus concentrations (Smil, 2000; Bartley et al., 2012), perhaps in part explaining why water bodies close to urban areas tended to have a higher probability of *mcyD* presence. In contrast, a negative correlation was observed between ammonia concentrations and *mcyD* presence in our dataset. High ammonia concentrations combined with strong sunlight (the latter being quite common in Israel and other semi-arid lands) have been shown to negatively affect *Microcystis* (Dai et al., 2012). Alternatively, the inverse correlation between *mcyD* presence and ammonia (as well as the inverse correlation with the dissolved ratio of nitrogen to phosphorus, Supplementary Figure 4A) may be due to nitrogen uptake by *Microcystis* and other phytoplankton. Unraveling the effect of different nutrients on *Microcystis* presence, bloom dynamics and toxicity is clearly required in order to ascertain which are the best methods to address toxic blooms (Paerl et al., 2014).

What causes some water bodies to have a higher pH, and how this relates to *mcyD* presence, is less clear. The measured high pH values may have been caused by CO_2_ depletion due to photosynthesis and thus may partly be indicative of high phytoplankton biomass (the samples were mostly collected around mid-day during the summer months). Indeed, pH and Chl *a* concentrations are somewhat positively correlated in our dataset (Supplementary Table 2). However, the pH of freshwater may also be affected by other parameters, such as the bedrock or sediment type and the concentrations and uptake kinetics of inorganic nitrogen compounds. The pH may also be affected by many types of organic and inorganic compounds, including some widely used by industry. Regardless of what causes the high pH, such conditions may select for *Microcystis* over other phytoplankton species, as they often have higher pH tolerance, and may also select for toxic over non-toxic *Microcystis* strains (Van de Waal et al., 2011).

An intriguing result of our analysis is that the distance from agricultural land does not emerge as an important predictor of *mcyD* presence in our dataset. Several studies have suggested that freshwater bodies with catchment areas comprising a high percentage of agricultural land tend to have higher cyanobacterial biomass (Katsiapi et al., 2012) as well as potentially higher microcystin levels (Beaver et al., 2014). Such a link between regional land use and algal or cyanobacterial biomass, however, may depend on the connectivity of the water bodies (i.e., to other water bodies through streams or rivers, Catherine et al., 2008; Lear et al., 2013) and on the ratio of the water body volume and the catchment area (Katsiapi et al., 2012). In Israel, most of the water bodies are isolated (not connected to a network). Additionally, many of the sampled water bodies receive multiple inputs, including precipitation runoff, groundwater, waste-water after different levels of treatment and spring water. These aspects may explain why agricultural land use seems to be not important for *mcyD* presence in our dataset. While our sampling was limited to Israel, a combination of similar hydrology and the presence of toxic *Microcystis* blooms is observed in many other Mediterranean and semi-arid regions, including much of the Middle East, Greece (e.g., Gkelis et al., 2015), Sicily (Naselli-Flores et al., 2007), Spain (Asencio, 2013), and parts of Australia (Sinang et al., 2013). Further, research is needed to determine to what extent our results can be generalized to these climatically- and hydrologically- similar regions.

Importantly, in this study we considered only the planktonic cells found in the surface water next to the shore, however, spatial heterogeneity in the distribution of phytoplankton may occur within a single water body, especially with floating organisms such as *Microcystis* which are strongly affected by wind conditions. Moreover, *Microcystis* may also survive extended periods of adverse conditions as dormant resting stages in the sediment, rapidly reviving and contributing to blooms (Ståhl-Delbanco et al., 2003; Cirés et al., 2013). Nevertheless, most of the locations that were *mcyD*-negative remained so 4 years later, suggesting that some locations are inherently less hospitable to the potentially-toxic *Microcystis* strains. Additional studies with better resolved land-use maps, sampling regimes incorporating also the sediment and following the same locations over time are required in order to determine how stable the observed patterns are.

### Do niche separation, non-random dispersal or biotic interactions underlie the preference of a toxinogenic *Microcystis* clade to fish ponds?

*Microcystis* is one of several globally abundant freshwater cyanobacteria that are able to colonize and flourish in a wide range of habitats (Wilson et al., 2005; Fan and Wu, 2012). In the model marine cyanobacterium *Prochlorococcus*, genetically and physiologically different ecotypes have evolved to live under different conditions, for example under high- and low light conditions (Biller et al., 2014). In contrast, previous studies have suggested that *Microcystis* populations are not organized in ecotypes (van Gremberghe et al., 2011; Humbert et al., 2013; Moreira et al., 2013), and that their success may be attributed to a large and highly plastic genomes. Such genomes encode many regulatory and metabolic genes, enabling rapid acclimation and adaptation to fluctuating conditions in “unstable” freshwater environments (Humbert et al., 2013). Nevertheless, we have observed that at least one clade of *Microcystis* strains, defined using the *mcyA* gene sequence, preferentially inhabits fish ponds and is under-represented in irrigation reservoirs (Figure 5). It is tempting to speculate that this clade is specifically adapted to fish ponds, representing the first example of niche specialization in *Microcystis*. However, other explanations may be given, for example, that the association of this clade with fish ponds is due to non-random dispersal. Specifically, most of the Israeli aquaculture system relies on hatching and growth of juvenile fish in a small number of dedicated facilities, with the fish then distributed as fingerlings to fish ponds. This could enable concurrent transfer of the hatcheries microbiota, including *Microcystis*, to the open fish ponds. Another potential vector for non-random *Microcystis* dispersal is with migratory birds, many of which visit multiple fish ponds on their annual return route from Africa to Europe (van Leeuwen et al., 2012; Moreira et al., 2014). The observed patterns could also represent historical distribution patterns, because established populations often have a competitive advantage over newly introduced migrants, a phenomenon termed the “priority effect” (Van Gremberghe et al., 2009). Finally, other biotic factors associated with fish ponds may serve to control the distribution of *Microcystis*. For example, some species of the dinoflagellate *Peridinium w*ere documented inhibiting *Microcystis* growth and reproduction (Wu et al., 1998; Vardi et al., 2002), and heterotrophic bacterial communities may also affect the structure of *Microcystis* populations (Choi et al., 2005; Shen et al., 2011; Zhang et al., 2011). Long term analysis of the presence and abundance of toxinogenic *Microcystis* species, combined with whole-population 16S and 18S analyses, may reveal co-occurrence patterns between *Microcystis* and other micro-organisms that will help test these hypotheses. In parallel, isolation and characterization of fish-pond associated strains may help determine whether such strains have growth advantages under conditions commonly found in fish ponds, or whether the changes in *mcyA* gene sequence are associated with differences in the microcystin molecule, as previously shown for the *mcyA* gene (Allender et al., 2009). Our observations of a clade of *Microcystis* associated with fish ponds could not be expanded using *mcyA* sequences found in public databases, since the vast majority of these sequences are not associated with any metadata describing the location from which they were isolated. This highlights the need for better association between sequence and environmental data in order to facilitate our understanding of the factors controlling the distribution and abundance of microbes in nature.

As the Earth's climate, population, water-, and land-use rapidly change, predicting when and where aquatic microorganisms, including *Microcystis* and other toxic phytoplankton, will live and bloom is critical in order to facilitate the design of cost-effective monitoring systems and management approaches to reduce human and environmental exposure. Integrating our understanding of the biology and ecology of *Microcystis* and other aquatic microbes into a framework that encompasses geography, land, and water-use (Catherine et al., 2008; Lear et al., 2013) is critical as we inch forward to understand how water resources and their biotic communities are shaped under anthropogenic pressures.

## Author contributions

SM and DS designed the research, SM, DA, MG, YY, and DS performed field sampling, SM, DA, and YY analyzed samples, LB formulated and performed the GLM analyses and MG performed the multivariate analyses. All authors analyzed the data and wrote the manuscript.

## Funding

This study was supported in part by grant 3-10342 from the Israel Ministry of Science and Technology and by Marie Curie Career Integration Grant MICROBES-2-MODEL.

### Conflict of interest statement

The authors declare that the research was conducted in the absence of any commercial or financial relationships that could be construed as a potential conflict of interest.
