# Supplementary material for: Distribution and Habitat Specificity of Potentially-Toxic Microcystis across Climate, Land, and Water Use Gradients
Source: Front Microbiol. 2016 Mar 15;7:271. doi: 10.3389/fmicb.2016.00271 (PMC4791393; doi:10.3389/fmicb.2016.00271)
Supplement: Supplementary file 1 [file DataSheet1.docx]

Supplementary Material

**Distribution and habitat specificity of potentially-toxic *Microcystis* across climate, land and water use gradients.**

Sophi Marmen^1^, Dikla Aharonovich^1^, Michal Grossowicz^1^, Lior Blank^2^, Yosef Z. Yacobi^3^ and Daniel Sher^1*^

***^1^*** *Department of Marine Biology, Charney School of Marine Sciences, University of Haifa, Haifa, Israel*

***^2^*** *Department of Plant Pathology and Weed Research, ARO, Volcani Center, Bet Dagan 50250, Israe*l

**^3^** *Israel Oceanographic & Limnological Research, Yigal Allon Kinneret Limnological Laboratory*

^*^Corresponding author: Dr. Daniel Sher, Department of Marine Biology, Leon H. Charney School of Marine Sciences, University of Haifa, Haifa 31905, Israel. email: [dsher@univ.haifa.ac.il](mailto:dsher@univ.haifa.ac.il)

**1. Supplementary methods**

**1.1 PCR program, primer sequences and cloning procedure**

All PCR reactions consisted of the following components: 10-50 ng μl^-1^ of template DNA, 12.5 μl MyTaq Red mix x2 buffer, 0.5 μl of 10 μM stock of forward and reverse primers in the final volume of 25 μl. The general PCR program was: 2 min 94°C, (30 sec 94°C, 30 sec 55°C, 1.5 min 72°C) X 37 cycles for mcyA/D and 30 cycles for 16S genes, 7 min 72°C, 2 min 4°C. DNA samples from toxic blooms in lake Deutsch-Baselitzer Grossteich, Germany, were used as a positive control (kindly provided by Dr. Hans-Peter Grossart, Leibniz-Institute of Freshwater Ecology and Inland Fisheries, Stechlin, Germany) for the amplification of mcy genes. McyA and mcyD amplicons were purified using Wizard® SV Gel and PCR Clean-Up System Kit (Promega, USA), and were cloned using pGEM®-T Easy Vector System Kit (Promega, USA). Purified plasmids were sent to Macrogen (Amsterdam, Netherlands) for sequencing.

**1.2 Generalized Linear Modeling**

Generalized Linear Modeling is a statistical linear expression used to model the relationship between a dependent variable and a set of independent variables (Dobson, 1990; McCullagh and Nelder, 1989). The purpose of a GLM analysis is to determine which independent variables affect the dependent variable and in what proportions. Multi-model inference based on the Akaike Information Criterion (AIC) was used to rank the importance of variables (Burnham and Anderson, 2002; Saltz, 2011) using the package “glmulti” in R (version 3.1.0) (Calcagno, 2010). This package models all possible combinations of the explanatory variables. Generally, comparing every possible model is not recommended as a model selection method (Burnham and Anderson, 2002). However, this approach can be a useful for assessing differences among models in case meaningful a priori model selection is not possible due to limited knowledge of species-habitat relationships (Doherty *et al*., 2012). The coefficients associated with each variable and their relative importance were assessed using a multi-model average. Evaluation of the relative importance of the explanatory variables individually was performed using the sum of the relative evidence weights for each model in which a given variable appears.

Notably, two variables (precipitation and elevation) strongly correlated with mdt8 (R>-0.8 and R>-0.7, respectively). In addition there was a certain level of geographic bias to the data related to the presence of Israel at the boundary of several different climatic regions (e.g. less agricultural land in the south of Israel, which lies in the global desert belt, Figure 1c), all of which presented challenges to the analysis (Supplementary Tables S3-4). Nevertheless, as presented in the results, several robust inferences (described in the main text) can be drawn from this analysis, and are supported by re-analysis of the data without the co-correlated factors and focusing only on the northern locations (supplementary tables S3-4, supplementary figure 5 ).

**2. Supplementary tables and figures**

**2.1 Supplementary tables**

**Supplementary Table 1:** PCR primers used in this study

| **Gene** | **Primer** | **Sequence (5'-3')** | **Product length (bp)** | **Reference** |
| --- | --- | --- | --- | --- |
| mcyA | MSF  MSR | ATCCAGCAGTTGAGCAAGC  TGCAGATAACTCCGCAGTTG | 1300 | Tillett *et al*., 2001 |
| mcyD | mcyDF2 mcyDR2 | GGTTCGCCTGGTCAAAGTAA  CCTCGCTAAAGAAGGGTTGA | 300 | Kaebernick *et al.,* 2000 |
| 16S (cyanobacteria) | CYA106F  CYA781R(a)  CYA781R(b) | CGGACGGGTGAGTAACGCGTGA  GACTACTGGGGTATCTAATCCCATT  GACTACAGGGGTATCTAATCCCTTT | 700 | Nübel *et al*., 1997 |
| 16S (general) | 27f-CM  1492r | AGAGTTTGATCMTGGCTCA  TACCTTGGTTACGACT | 1400 | Frank *et al*., 2008 |

**Supplementary Table 2:** Cross-correlation values for individual parameters used in the GLM analysis.

**Supplementary Table 3:** Coefficients of each of the local and regional parameters in the GLMs using all data, removing major cross-correlating parameters and using the locations in the north of Israel only. The following abbreviations are used: mdt8 – mean temperature during August; distReserve – distance from nature reserves; distAgri – distance from agricultural land; distBuilt – distance from urban areas.

**Supplementary Table 4:** Importance values of each of the local and regional parameters in the GLMs using all data, removing major cross-correlating parameters and using the locations in the north of Israel only. Parameters with importance >0.5 are highlighted. The following abbreviations are used: mdt8 – mean temperature during August; distReserve – distance from nature reserves; distAgri – distance from agricultural land; distBuilt – distance from urban areas.

**2.2 supplementary figures**

**
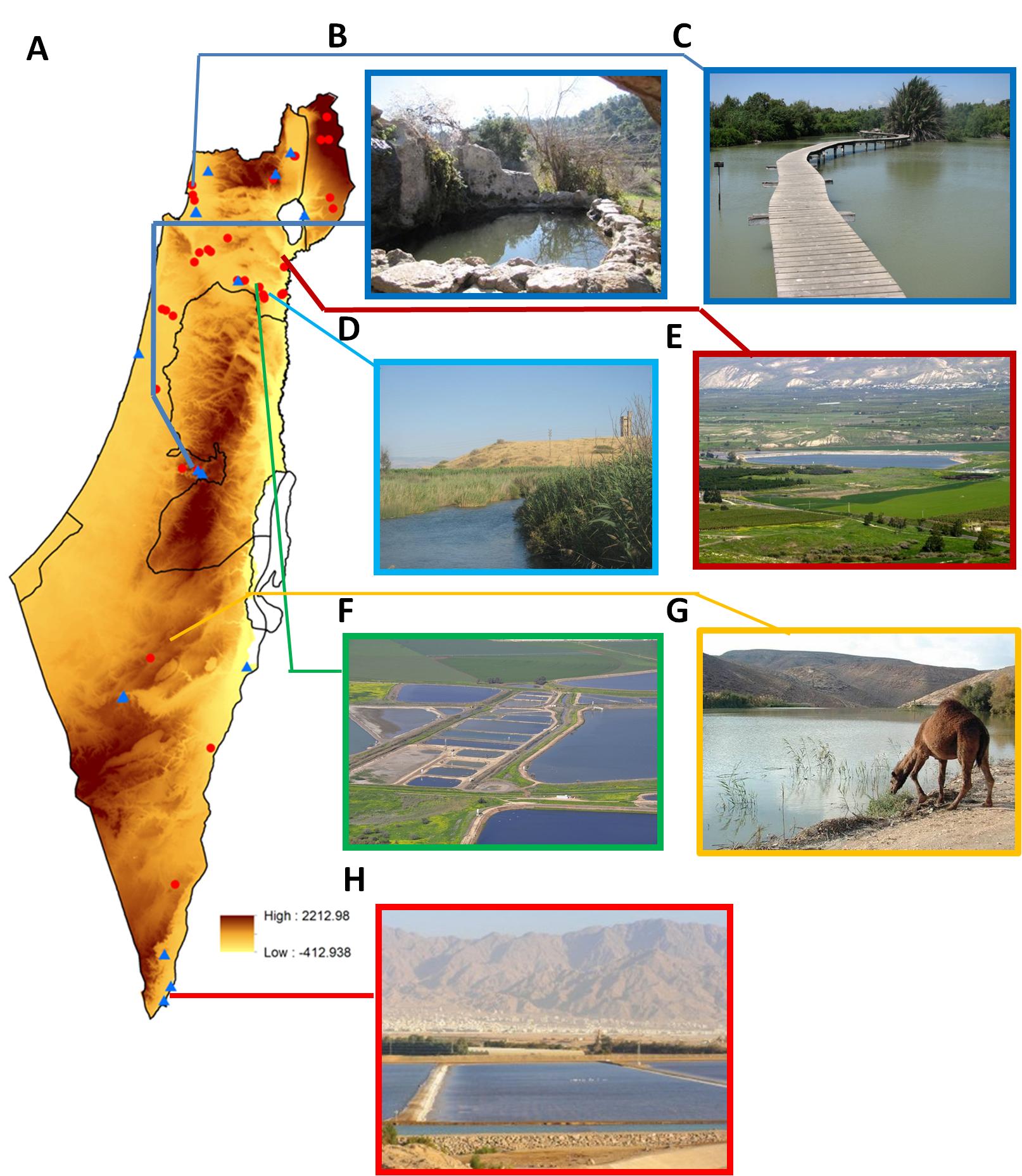
**

**Supplementary Figure 1: Brief descriptions of the various types of water bodies sampled.** (A) A physical (elevation) map of Israel, with the sampling locations represented by red (mcyD positive) and blue (mcyD negative) dots. The names in the following panels refer to sampling locations (see supplementary excel file). (B, C) Springs and spring systems (Ein Sarig and Ein Afek, respectively). Many small springs in Israel are caught in man-made pools which are typically several square meters in area and 1-2 meters in depth, and may be dry for part of the year. Many of these pools were used for irrigation until the mid-20th century, and are now occasionally used as water sources for herds or for recreation. There are several larger spring systems, where larger amounts of water emerge, typically from several close sources, flowing into larger pools. Most such spring systems are protected as nature reserves or natural parks. (D) Rivers tend to be relatively small (better described as creeks in other climatic regions), typically 2-4 meters wide and no more than 1-2 meters deep. Depending on their water source and location they may be protected (e.g. Kibbutzim River, depicted in panel D) or contaminated to various levels with wastewater from different sources. (E) Irrigation reservoirs are very heterogeneous, collecting water from many different sources: rainwater and agricultural runoff, wastewater (at various levels of treatment) and water pumped from aquifers. These reservoirs are then used for agriculture, and occasionally are also stocked with fish. The image shown is of the Gesher reservoir. F) Fish ponds in the Harod valley (line 29-40 in the Sup Excel file). These ponds, as well as others sampled around Israel, are used for intensive aquaculture rearing, often of carp (Cyprinus carpio) or tilapia (Oreochromis niloticus). The origin of the water is typically from springs or aquifers. T G) Lakes were operationally defined as larger water bodies not used for irrigation or aquaculture. A photo is shown of Lake Yeruham in the south of Israel, where water (primarily from desert flash-floods) is dammed and the resulting lake used for recreation.. H) Salterns are used for the production of salt from water from the Red Sea. The salterns near Eilat are rich in plankton and are often visited by large flocks of migrating flamingoes. We thank the following people for the photos shown: panels B-D - Amit Mendelsson; E - Avi Hirschfeld (JNF); F - Yaron First; G - Dubi Zakai; H - Amir Gur.


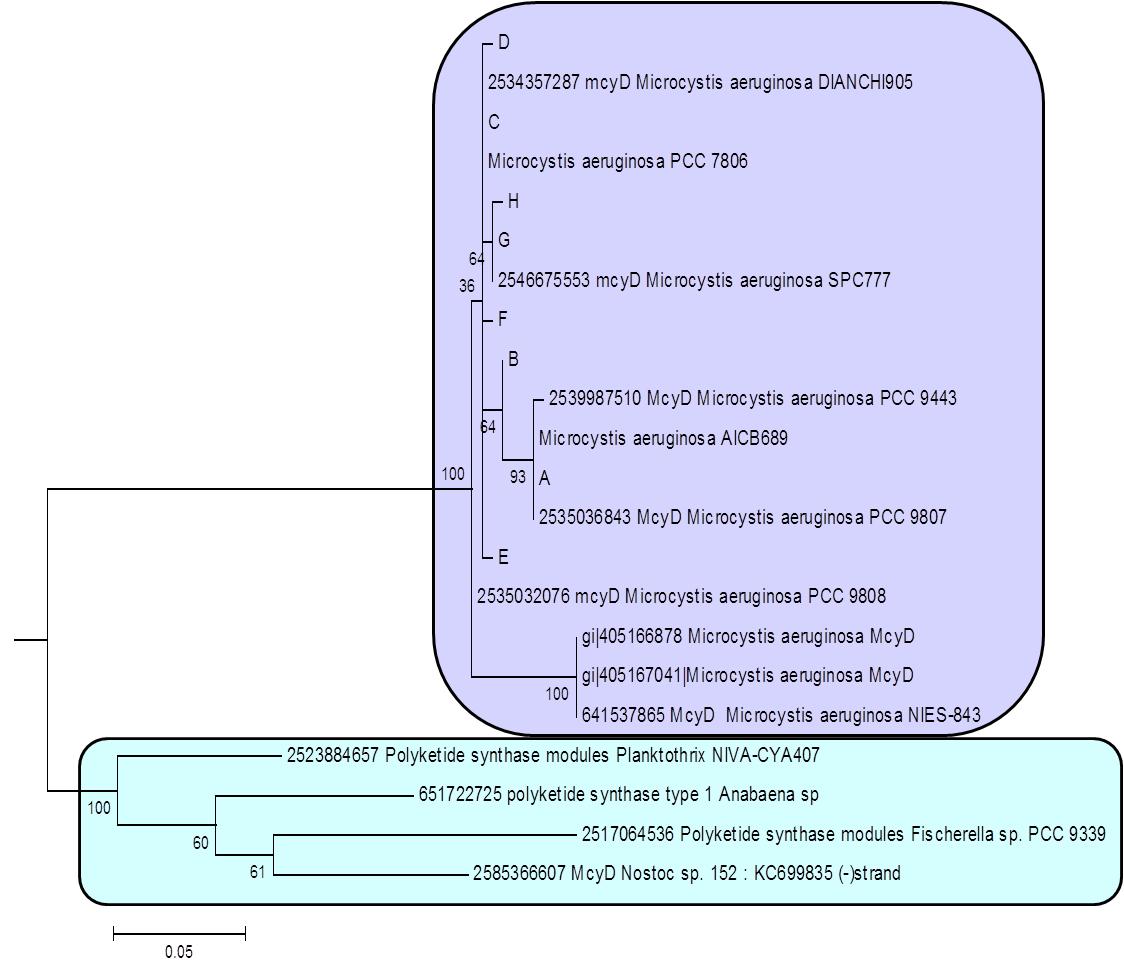


**Supplementary Figure 2: A maximum likelihood phylogenetic tree of *mcyD* genes from sequenced genomes and from our study.** Eight cloned sequences marked as A-F cluster with *mcyD* genes from genomes of *Microcystis aeruginosa,* and are clearly separated from *mcyD* genes from the genomes of other cyanobacteria. This indicates the ability of the primers to amplify the relevant genes from *Microcystis* but not from other cyanobacterial genera in our samples. The tree was rooted at midpoint.


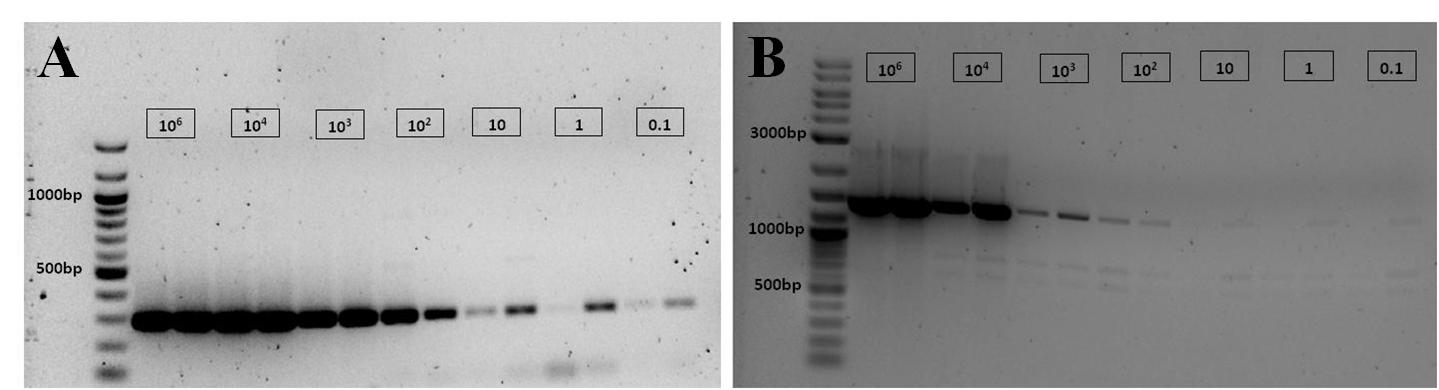


**Supplementary Figure 3:** **Sensitivity of the PCR primers for the *mcyD* and *mcyA* genes.** Known numbers toxinogenic *Microcystis aeruginosa* cells (MGK strain, Schatz *et al.,* 2005, isolated by Dr. Ora Hadas and kindly provided by Dr. Assaf Sukenik) were mixed with cultures of *Tetraselmis suecica*, strain K-0297 (Scandinavian Culture Collection of Algae and Protozoa). The final mixture contained the following toxic cells numbers: 10^5^, 10^3^, 10^2^, 10, 1, 0.1, 0.01 cells ml^-1^. Ten ml of the final mixed cultures were filtered onto GF/F filters followed by DNA extraction and PCR amplification as described in the materials and methods. Numbers above the bends represent the number of toxic cells/ filter. Consistant amplification can be observed from 10 cells/filter for *mcyD* gene (a), and 10^2^ cells/filter for *mcyA* gene (b).

**
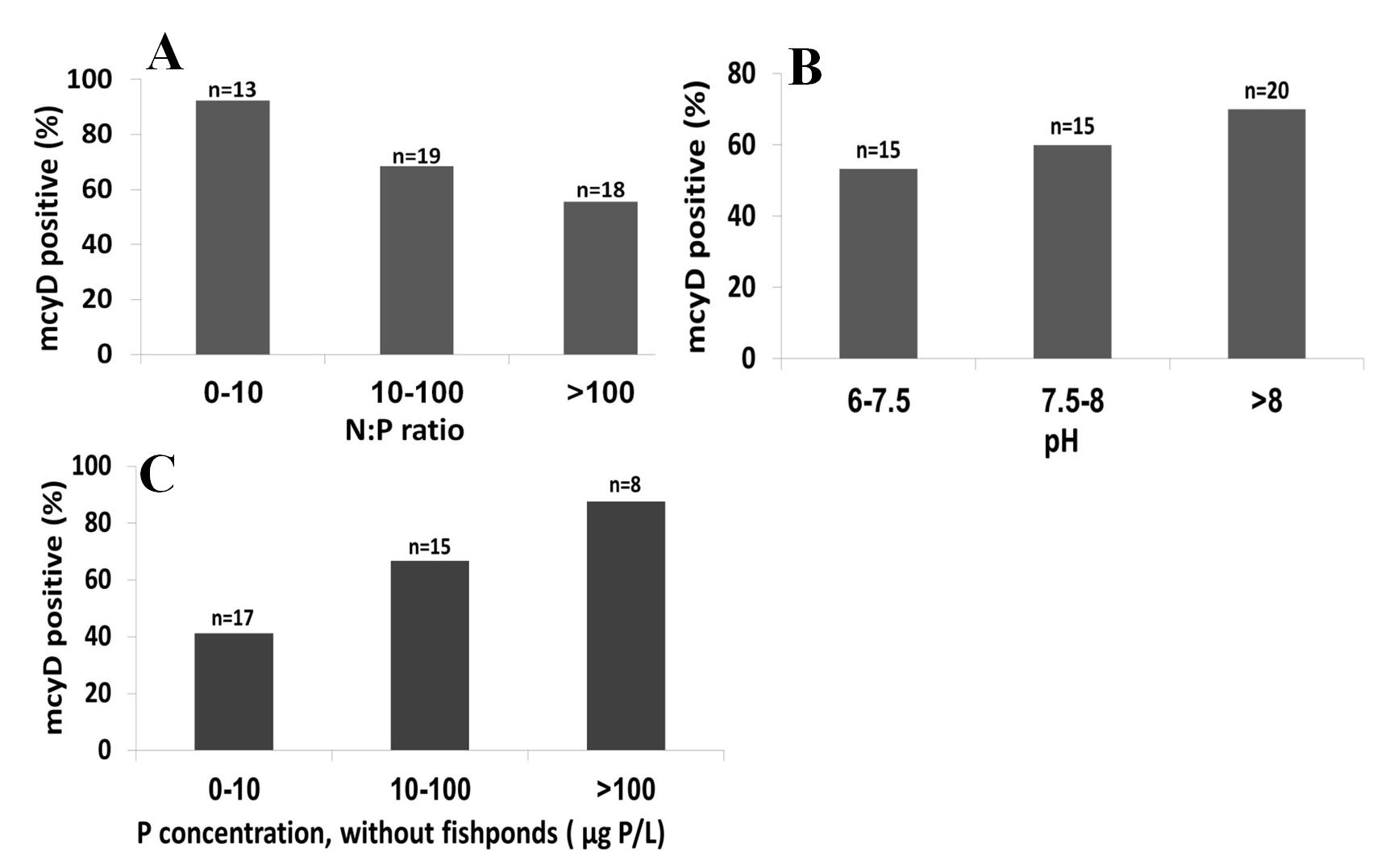
**

**Supplementary Figure 4. Trends of presence of *mcyD* gene across different a- biotic parameters.** Several of the parameters measured (nutrient concentrations and pH) exhibited different trends with the presence of *mcyD* gene but these were not statistically significant. These include a trend of high % *mcyD* presence in water with low total N: total P ratio (a); and high % *mcyD* presence in water with high pH values (b). Measurement of *mcyD* positive percent without including samples from fish ponds revealed also a positive trend (c). The correlation analyses were done using χ2 test with p < 0.05.

**
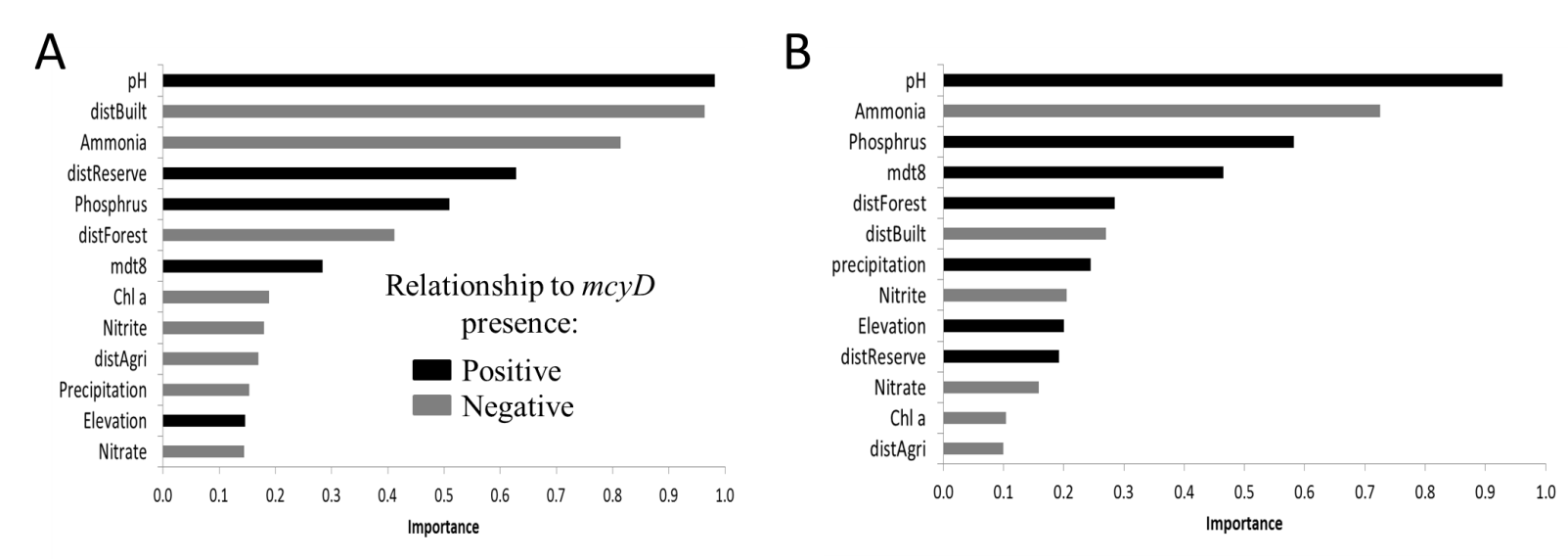
**

**Supplementary Figure 5. The relative importance of different local and regional parameters in a Generalized Linear Model (GLM) of the presence/absence of *mcyD* genes, with and without the sampling locations in the desert south of Israel.** Panel A presents the analysis with all the locations (same as in Figure 4), whereas panel B presents the results with the locations in the desert south of Israel removed. In both analyses, pH phosphorus concentrations are positively related to mcyD presence, and ammonia is negatively related. The main differences are in the importance of temperature (mdt8), which is larger when the southern locations are excluded, and the lower importance of regional characteristics such as the distance from built areas, reserves and forests. This is likely due to the much lower densities of these parameters in the desert (see figure 1C).
